# Acquisition of Cross-Resistance to Bedaquiline and Clofazimine following Treatment for Tuberculosis in Pakistan

**DOI:** 10.1128/AAC.00915-19

**Published:** 2019-08-23

**Authors:** Arash Ghodousi, Alamdar Hussain Rizvi, Aurangzaib Quadir Baloch, Abdul Ghafoor, Faisal Masood Khanzada, Mehmood Qadir, Emanuele Borroni, Alberto Trovato, Sabira Tahseen, Daniela Maria Cirillo

**Affiliations:** a Emerging Bacterial Pathogens Unit, Division of Immunology, Transplantation and Infectious Diseases, IRCCS San Raffaele Scientific Institute, Milan, Italy; b National TB Reference laboratory, National TB Control Program, Islamabad, Pakistan; c National TB Control Program, Islamabad, Pakistan

**Keywords:** bedaquiline, clofazimine, whole-genome sequencing, XDR, antibiotic resistance, multidrug resistance, tuberculosis

## Abstract

We report on the first six cases of acquired resistance to bedaquiline in Pakistan. Seventy sequential isolates from 30 drug-resistant-tuberculosis patients on bedaquiline-containing regimens were retrospectively tested for bedaquiline resistance by MIC testing and by the detection of mutations in relevant genes. We documented cases failing therapy that developed specific mutations in *Rv0678* and had increased MICs associated with cross-resistance to clofazimine during treatment. This study underlines the relevance of surveillance programs following the introduction of new drugs.

## Abstract (precis)

We report on the first six cases of acquired resistance to bedaquiline in Pakistan. Seventy sequential isolates from 30 drug-resistant-tuberculosis patients on bedaquiline-containing regimens were retrospectively tested for bedaquiline resistance by MIC testing and by the detection of mutations in relevant genes. We documented cases failing therapy that developed specific mutations in *Rv0678* and had increased MICs associated with cross-resistance to clofazimine during treatment.

## TEXT

In 2018, based on an evidence-based policy, the World Health Organization (WHO) updated its guidelines on the treatment of drug-resistant tuberculosis (TB) (1). The new guidelines recommend bedaquiline as a core drug in the standard combination regimen for the treatment of rifampin-resistant TB. As a result, the number of patients eligible to receive bedaquiline-containing regimens will significantly increase. Encouraging results from studies on the use of bedaquiline in all-oral shorter-course regimens for the treatment of rifampin-resistant TB will also result in an even greater use of bedaquiline by TB control programs (2–4). The scale-up of bedaquiline use should prompt, in addition to extensive pharmacovigilance to monitor adverse effects (5), the development of the capability to rapidly identify the emergence of resistance in patients during therapy.

In the present study, we report on the first six cases of acquired bedaquiline resistance identified in Pakistan. The study was conducted within the framework of a retrospective surveillance project to monitor the acquisition of resistance to bedaquiline implemented by the National TB Reference Laboratory (NRL) in Pakistan and TB Supranational Reference Laboratory (SRL-Milan). Bedaquiline was introduced in Pakistan in November 2015 for the treatment of multidrug-resistant/extensively drug-resistant (MDR/XDR) TB. Since then, all clinical isolates from culture laboratories serving MDR patients on bedaquiline-containing regimens enrolled at six sites located in six different cities were sent to the NRL in Islamabad for drug susceptibility testing. For 30 patients, cultures remained positive over time. The baseline and one or more available follow-up isolates collected from these 30 patients during treatment were shipped to the SRL-Milan, between November 2017 and May 2018, to perform MIC testing for bedaquiline and genomic analysis by whole-genome sequencing (WGS). Patients’ clinical data were retrospectively extracted from medical records. The institutional review board for the HIV, TB, and Malaria program, Pakistan, approved this study.

At SRL-Milan, all strains underwent MIC testing for bedaquiline in 7H11 medium, and for all isolates showing an increased MIC compared to the baseline isolate, further MIC testing for bedaquiline and clofazimine was conducted using the Bactec MGIT960 (BD, Franklin Lakes, NJ, USA) (6). The H37Rv (ATCC 27294) strain was used as a susceptible control in MIC testing. Bedaquiline dry powder was supplied by Janssen-Pharmaceutica (Beerse, Belgium). WGS was carried out with the Illumina Nextera-XT DNA sample preparation kit to prepare paired-end libraries of 150-bp read length to sequence on an Illumina NextSeq platform. Data analysis and single nucleotide polymorphism (SNP) calling were performed using the MTBseq-Pipeline on low-frequency detection mode (7). Genes associated with resistance to bedaquiline and/or clofazimine (*atpE*, *Rv0678*, *pepQ*, and *Rv1979c*) were screened for mutations (8–11).

**FIG 1:**
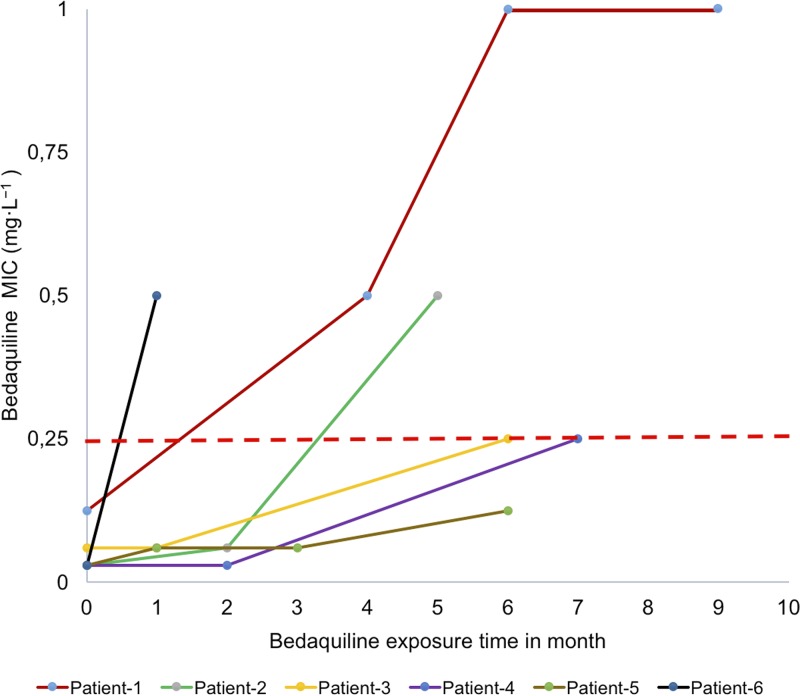
The MICs of bedaquiline performed in 7H11 medium in patients under treatment with bedaquiline-containing regimens at different time points. The dashed line in red shows the currently identified critical concentration for bedaquiline in 7H11 medium by the WHO (13).

We studied 70 isolates from patients (*n* = 30; 8 MDR, 15 pre-XDR, and 7 XDR) enrolled in bedaquiline-containing regimens. The sequential isolates tested included two strains from 22 patients, three from 6 patients, and four from 2 patients. All baseline strains included in the study were sensitive to bedaquiline. Six patients developed an increase in bedaquiline MICs in 7H11 medium (range, 0.125 to >0.5 mg·liter^−1^) during therapy, and five of them became resistant to bedaquiline according to the current critical concentration proposed for the drug (Fig. 1). The phenotypic/genotypic characteristics of Mycobacterium tuberculosis isolates from these six HIV-negative, unrelated pulmonary TB patients are summarized in Table 1.

**TABLE 1:** The phenotypic/genotypic characteristics of M. tuberculosis isolates from the six patients who acquired cross-resistance to bedaquiline and clofazimine

Strain or patient ID^*a*^	Previous second-line treatment regimen/outcome^*b*^	DST profile at start of BDQ-containing regimen	BDQ exposure (mo)	M. tuberculosis strain testing results										Treatment outcome
				pDST results for MIC (mg·liter^−1^) for:			Genome coverage (×)	WGS^*c*^						
				BDQ in 7H11	BDQ in MGIT	CFZ in MGIT		*Rv0678*	*pepQ*	*atpE*	*Rv1979c*	NCBI accession no.	Lineage	
Strain														
H37Rv (NC_000962.3)				0.03	0.25	0.25	43.28	WT	WT	WT	WT			
Patients														
1	NO	Pre-XDR	0	0.125	0.5	0.5	71.67	WT	WT	WT	WT	SRR9028486	Delhi-CAS(3)	Failure
			4	0.5	Not tested	Not tested	40.57	779130_141-142_Ins-C	WT	WT	WT	SRR9028477		
			6	0.5	Not tested	Not tested	58.11		WT	WT	WT	SRR9028476		
			9	>0.5	4	4	68.20		WT	WT	WT	SRR9028485		
2	Am(8), Lfx, Cs, Eto, Z, E, B6/failure	MDR	0	0.03	0.5	0.5	80.97	WT	WT	WT	2221732_P478G	SRR9028481	Delhi-CAS(3)	Cured
			2	0.06	Not tested	Not tested	127.50	WT	WT	WT		SRR9028488		
			5	0.5	4	4	90.23	779130_141-142_Ins-C	WT	WT		SRR9028489		
3	Am, Lfx, Eto, CS, Z, PAS, B6/failure	MDR	0	0.06	0.25	0.5	96.26	WT	WT	WT	WT	SRR9028483	Delhi-CAS(3.1.2)	Failure
			1	0.06	Not tested	Not tested	86.92	WT	WT	WT	WT	SRR9028490		
			6	0.25	2	2	100.78	779048_V20G	WT	WT	WT	SRR9028494		
4	Cm(12), Mfx, Cs, Eto, Cfz, Z, Amx-clv/failure	XDR	0	0.03	0.25	0.5	81.93	WT	WT	WT	WT	SRR9028487	Delhi-CAS(3)	Still on treatment
			2	0.03	Not tested	Not tested	111	WT	WT	WT	WT	SRR9028484		
			7	0.25	2	4	102.74	779127_138-139_Ins-G	WT	WT	WT	SRR9028491		
5	Cm(12), Mfx, Cs, Eto, Cfz, Z, PAS, Clr, Amx-clv, H, E, B6/failure	XDR	0	0.03	0.5	0.5	68.40	WT	WT	WT	WT	SRR9028482	Delhi-CAS(3)	Failure
			1	0.06	1	Not tested	125.22	WT	WT	WT	WT	SRR9028493		
			3	0.125	2	Not tested	116.61	779127_138-139_Ins-G	WT	WT	WT	SRR9028480		
			6	0.125	2	4	109.03		WT	WT	WT	SRR9028478		
6	Cm(12), Mfx, Cs, Eto, Cfz, LNZ, PAS, Z, Amx-clv, B6/failure	XDR	0	0.03	0.5	1	99.35	WT	WT	WT	WT	SRR9028479	Euro-American -4.9	Failure
			1	0.5	4	4	83.34	779181_192-193_Ins-G	WT	WT	WT	SRR9028492		

a ID, identifier.

b AM, amikacin; Amx-clv, amoxicillin-clavulanic acid; Cfz, clofazamine; Cm, capreomycin; Cs, cyclocerine; E, ethambutol; Eto, ethionamide; Lfx, levofloxacin; LNZ, linozolid; Mfx, moxifloxacin; PAS, para-aminosalicylic acid; B6, vitamin B6; Z, pyrazinamide. Numbers in parentheses indicate the duration of the administration of the antibiotic in months.

c WT, wild type.

Patient 1, a newly diagnosed rifampin-resistant TB case, was enrolled in second-line treatment. The patient remained culture positive and was subsequently diagnosed as a pre-XDR TB case, and after 5 months of second-line treatment, bedaquiline, clofazimine, and linezolid were added to the regimen. The patient continued to be culture positive, and 6 months later, delamanid was also added to the regimen; ultimately, treatment failure was declared. The first increase in bedaquiline MIC was seen at the fourth month of treatment (0.5 mg·liter^−1^ in 7H11) concomitantly with WGS analysis showing an insertion (Ins) at nucleotide position 141 to 142 of *Rv0678* (genomic position 779130). At month 9, the isolate became resistant to delamanid (MIC >1 mg·liter^−1^) (data not shown). WGS analysis confirmed the presence of the *Rv0678* mutation and also showed a mutation in *fgd1* (G104S), probably responsible for delamanid resistance.

Patients 2 and 3 had MDR TB with a history of failed second-line treatment taken for 20 and 17 months, respectively, both were then re-enrolled on a bedaquiline- and clofazimine-containing regimen. For both patients, no difference was seen in the bedaquiline MIC between the baseline isolate and the isolates collected after two and one month of bedaquiline-containing treatment. A subsequent isolate from patient 2 at the fifth month of treatment became resistant to both bedaquiline and clofazimine, and WGS analysis showed the appearance of an insertion at position 141 to 142 of *Rv0678*. Similarly, isolate from patient 3 after 6 months of bedaquiline showed 8- and 4-fold increases in bedaquiline and clofazimine MICs in mycobacterial growth indicator tubes (MGITs), and a V20G mutation in *Rv0678* was detected. Moreover, the M. tuberculosis strains from patient 2 at baseline and at two and five months of treatment carried a P478G mutation in *Rv1979c*, suggesting that this mutation has no effect on clofazimine resistance.

Patients 4, 5, and 6 were XDR TB cases failing a second-line treatment regimen containing clofazimine administered for 20, 14, and 12 months, respectively. Baseline isolates of all three cases had a bedaquiline MIC of 0.03 mg·liter^−1^ in 7H11 and showed no detectable mutations in *Rv0678*, *pepQ*, *atpE*, and *Rv1979c*. The follow-up isolates from patients 4 and 5 collected at months 6 and 5 during treatment showed an increase in bedaquiline and clofazimine MIC associated with an insertion at position 138 to 139 of *Rv0678* (genomic position 779127). Patient 6 showed a substantial increase in MICs for both bedaquiline and clofazimine (8-fold for bedaquiline, 4-fold for clofazimine) after 1 month of therapy, and we detected an insertion in *Rv0678* at a variant frequency of 12.7% that was not present at baseline. This patient ultimately failed treatment.

Breakpoints for bedaquiline are still provisional (12, 13), and patients are started on treatment without susceptibility tests. We adopted 0.25 mg·liter^−1^ as the susceptibility breakpoint in 7H10 and 7H11 (14, 15), which is above the MIC that inhibits 90% of the isolates or strains (0.125 mg·liter^−1^). However, with this breakpoint, the strains collected from two patients reported as “failures” at the end of treatment (patient 4, MIC of 0.25 mg·liter^−1^, 8-fold higher than baseline; and patient 5, MIC of 0.125 mg·liter^−1^, 4-fold higher than baseline) would have been categorized as susceptible. This finding indicates that monitoring MICs during treatment could be a better predictor for failure than single testing at the critical concentration. The genetic basis of resistance to bedaquiline is still the subject of much uncertainty. WGS analyses in different studies showed that bedaquiline-clofazimine cross-resistance arises through mutations in *Rv0678* (8, 9, 10) and *pepQ* (11). In this study, we show that the increase in the MICs to bedaquiline and clofazimine could be explained by mutations in *Rv0678* emerging during therapy. As reported in Table 1, we observed four different mutations, and three of these (138-139_Ins-G, 141-142_Ins-C, and 192-193_Ins-G) were previously reported as associated with bedaquiline resistance in M. tuberculosis clinical strains (10, 16). We show that the same mutations are associated with clofazimine resistance. As a result, regimens that contain both drugs might have to be reconsidered when these mutations are identified in order to reduce the risk of treatment failure for patients and the transmission of such strains in the community. Altogether, these data show that resistance to bedaquiline emerges during treatment and emphasize the importance of using MIC coupled, whenever possible, with WGS in national programs implementing bedaquiline for the treatment of MDR/XDR TB to monitor the emergence of resistance. Moreover, the collection of genomic data on mutations associated with bedaquiline and clofazimine resistance is crucial to lead future development of tools for fast detection of resistance. Introducing drugs without proper diagnostics to monitor drug resistance may lead to the amplification of hardly treatable cases.

## 

### Accession number(s).

Raw sequencing reads have been deposited at BioProject at NCBI (project accession number PRJNA540911).
